# Infection and Infertility

**DOI:** 10.1155/S1064744993000134

**Published:** 1993

**Authors:** Sebastian Faro

**Affiliations:** Department of Gynecology and Obstetrics University of Kansas School of Medicine 3901 Rainbow Blvd Kansas City KS 66160-7316 USA

## Abstract

Asymptomatic infection appears to be a common cause of fallopian tube damage resulting in ectopic pregnancy or infertility.


Infectious Diseases in Obstetrics and Gynecology 1:51–57 (1993)
© 1993 Wiley-Liss, Inc.

### Infection and Infertility

**Sebastian Faro**

*Department of Gynecology and Obstetrics, University of Kansas School of Medicine, Kansas City, KS*

ABSTRACT

**Asymptomatic infection appears to be a common cause of fallopian tube damage resulting in ectopic pregnancy or infertility.** © 1993 Wiley-Liss, Inc.

KEY WORDS

Bacterial vaginosis, *Mycoplasma*, *Ureaplasma*, asymptomatic infection

The role of serious pelvic infection in female infertility is well recognized. However, the role of asymptomatic infection caused by *Neisseria gonorrhoeae* and *Chlamydia trachomatis*, although of great concern, has not been well established. Significant research efforts have been made to define these asymptomatic sexually transmitted diseases (STDs) and their role in infertility. In conjunction with much of the theorizing on the infectious etiology of infertility, the role of the vaginal microflora has also been implicated in these infections. Currently, the initial infection is thought to be due to the gonococcus and/or chlamydia. This initial infection is then followed by ascension of potentially destructive microbes endogenous to the lower female genital tract.

### THE VAGINAL MICROFLORA

The lower female genital tract is a delicate ecosystem maintained in dynamic equilibrium. This balance can be tipped by any number of factors, both endogenous and exogenous. The bacteriologic make-up of the lower genital tract includes both synergistic bacteria and antagonistic organisms. The healthy vaginal ecosystem is characterized by a pH of 3.8 to 4.2; a slate-grey to white, odorless discharge; and the presence of other gram-positive commensal bacteria, with the dominant bacterium being *Lactobacillus*. Also present are many other bacteria, gram-positive aerobes, facultative gram-negative bacteria, anaerobic gram-negative bacteria, and gram-positive bacteria. The presence of *Lactobacillus* appears to be pivotal in maintaining the equilibrium and preventing potentially pathogenic bacteria from gaining dominance.[1] The current theory is that the production of lactic acid by lactobacilli maintains the appropriate pH. In addition, these bacteria produce hydrogen peroxide that is toxic to anaerobic bacteria, which do not produce peroxidase. When the balance is upset, the commensal bacteria decrease in number, reducing the hydrogen ion concentration and hydrogen peroxide and allowing the growth of facultative and anaerobic bacteria, thereby resulting in an unhealthy state. Important to remember is that, despite the many forms of vaginitis, certain types are more conductive to the production of upper genital tract infection, such as endometritis and salpingitis. The most common types of vaginitis are bacterial vaginitis (30% to 35% of cases), yeast vaginitis (20%–25%), and trichomoniasis (10%–15%).[2–4] Trichomoniasis should serve as a marker organism for the possible existence of other infections, as it is commonly found in association with gonorrhea and chlamydia.[5–7]

The unhealthy or abnormal vaginal flora is characterized by a pH >4.5. A discharge is also characteristic; it is usually dirty-grey but may be colors

**Address reprint requests to Sebastian Faro, M.D., Ph.D., Department of Gynecology and Obstetrics, The University of Kansas School of Medicine, 3901 Rainbow Blvd., Kansas City, KS 66160-7316.**

Received January 20, 1993
Accepted April 7, 1993
Review Article other than white. It may or may not be frothy and may or may not be homogenous. Typically, it emits an amine odor when mixed with 10% potassium hydroxide (KOH). Microscopic examination usually reveals the squamous epithelial cells to be covered by adherent bacteria, obliterating the cytoplasmic membrane and nucleus. In the case of bacterial vaginosis (BV), white blood cells (WBCs) are usually absent, thus the use of the suffix "-osis" to indicate the absence of an inflammatory reaction, but numerous individually free-floating bacteria are present.

Microbiologic evaluation of this type of lower genital tract will reveal the presence of large numbers of facultative and obligate anaerobes. With BV, the patient is more likely to have an STD. In one study, 54% of the women with BV reported having had an STD.[8] Other bacterial derangements of the vaginal microflora may exist, especially in patients who have been repeatedly treated for vaginitis but whose flora has not regained the composition of a healthy state. *Gardnerella vaginalis* vaginitis, originally described by Gardner and Dukes, consists of a vaginal discharge resembling that of BV, but differing in that the free-floating bacteria found in the vaginal fluid are not individually distributed but in aggregates. Rarely, if ever, are WBCs present.[9] Some patients present with bacterial vaginitis consisting predominantly of *Gardnerella vaginalis* with few, if any, other types of bacteria and, rarely, anaerobes. Whereas other types of vaginitis may have clinical parameters similar to those found with BV, 30% of patients with a discharge that liberates a foul odor will have BV and 17% of patients with clue cells but no dominant anaerobic bacteria will have BV.[10] In one study, patients with persistent vaginitis who had been repeatedly treated with antibiotics were enrolled.[11] All patients had a vaginal pH of $\geq 4.5$ and the liberation of amines when the discharge was mixed with 10% KOH. These patients were divided into two groups. The first group consisted of 48 patients. Microscopic analysis of the vaginal fluid of all patients in the first group demonstrated numerous individually free-floating bacteria and numerous WBCs. Half of the patients in this first group did not demonstrate *Gardnerella vaginalis* as part of the microflora, and half demonstrated *Gardnerella vaginalis* in counts of $<10^3$ colony-forming units per ml (cfu/ml) of vaginal fluid. The second group of patients all demonstrated *Gardnerella vaginalis*, but in high colony counts of $3 \times 10^5$ to $9 \times 10^9$ cfu/ml of vaginal fluid. The vaginal fluid of the patients in this second group revealed clue cells, rare WBCs, and bacteria floating in clumps, not individually. The finding of varieties of bacterial infections raises the concern regarding not the particular type of vaginitis, but the possibility that the bacteria present may ascend to the upper genital tract and act synergistically in causing a progressive infection.

Important to remember is that the individual with bacterial vaginitis or BV is at greater risk for having an accompanying STD. This epidemiologic information is important for two reasons. First, it implies that the patient's behavior pattern may currently place her at risk for upper genital tract infection. Second, it encourages the physician to obtain a detailed sexual history to determine if the patient has been at risk for a prolonged time, making the possibility of a past or present upper genital tract infection more of a reality.

### EVALUATION OF THE PATIENT FOR INFECTION

The physician should encounter little difficulty in evaluating the patient with an acute symptomatic genital tract infection. However, the goal should be the prevention of damage of the fallopian tubes which might result in abnormal function or infertility. The annual ectopic pregnancy rate from 1970 through 1989 has risen four-fold from 4.5 to 16.1/1000 pregnancies.[12] Hospital admissions for ectopic pregnancy in 1991 were 88,400, a 10% increase over 1988. This rise in the ectopic pregnancy rate is believed to be due to the rise in the number of cases of pelvic inflammatory disease (PID). Although obtaining data with regard to infertility and infection is difficult, the estimation has been made that, after a single episode of PID, approximately 12% of the women will be infertile; after two episodes, 25% will be infertile; and, after three or more episodes, more than 50% will be infertile.[13] The Centers for Disease Control has estimated that four million initial visits to physicians for PID were made in 1991, generating approximately 250,000 hospital admissions.[14]

To prevent fallopian tube damage, the physician must recognize the earliest stage of the disease process. The initial site of infection in the female patient is the cervix. However, the difficulty lies in the fact that the greatest percentage of infected women are asymptomatic. The physician may be clued in by asking the patient the appropriate questions indicating historical evidence for the possible existence of an STD:

1. Have you noticed a change in your vaginal discharge?
2. Do you have spotting following sexual intercourse?
3. Have you noticed a sudden onset of pain with sexual intercourse?

Close attention should be paid to the endocervical epithelium, specifically noting the presence of hypertrophy. A dacron swab should be passed into the endocervical canal and rotated for 5 to 10 seconds to determine if any endocervical mucus is present. Typical findings suggesting cervical infection, namely *Chlamydia trachomatis* and *Neisseria gonorrhoeae*, are that (1) the endocervical tissue bleeds easily when the pap smear is taken and (2) the patient's pap smear reveals inflammation.[15,16] The physician should remember that patients do not present with complaints of cervicitis and that cervical infection by *N. gonorrhoeae* and/or *C. trachomatis* is frequently asymptomatic.[16] Therefore, to prevent significant infection, the physician must be able to recognize early asymptomatic infection.

Infection of the fallopian tubes is thought to occur by progressive upward migration from the endocervix along the columnar epithelium lining the endocervix and endometrium. Patients who develop endometritis may not have overt signs or symptoms of infection. The patient may develop vague lower abdominal cramping-like pain, recent onset of dyspareunia or dysmenorrhea, breakthrough bleeding if she is utilizing oral contraceptive pills (OCPs) for birth control, or irregular bleeding if she is not using hormonal contraception.[17] Thus, the patient who is suspected of having endometritis should be thoroughly evaluated. Specimens for culture of *N. gonorrhoeae* and *C. trachomatis* should be obtained from the endocervix. Then, the portio of the cervix should be thoroughly cleansed. If Betadine is used, the cervix should be gently wiped off before an endometrial sample is taken. The endometrial sample is best obtained by inserting a pipette through the endocervical canal to the uterine fundus and obtaining a tissue specimen. The pipette should be carefully withdrawn without touching the vaginal walls. The specimen should be divided into two portions. One should be sent for histologic evaluation and the other one sent in an anaerobic transport vessel for the culture of *N. gonorrhoeae*, *C. trachomatis*, aerobic, facultative, and obligate anaerobic bacteria. The presence of plasma cells, especially if *C. trachomatis* is the infecting organism, is highly correlated with salpingitis.[18,19] Endometritis, frequently asymptomatic, has been reported to occur in approximately 40% of women with cervicitis.[18] It has also been reported that women clinically diagnosed as having salpingitis, but laparoscopically found to have normal fallopian tubes, had endometritis by biopsy.[20]

### RISK FACTORS

The first line of intervention is prevention; however, the physician rarely has the opportunity to teach prevention. Therefore, the existence of infection of the cervix or endometrium should be considered as the second line of intervention. The second opportunity to prevent serious tubal damage requires recognizing the patient at risk. The physician must be comfortable with taking a detailed sexual history from a young patient who is sexually active, especially one utilizing OCPs, Norplant (Wyeth-Ayerst Laboratories, P.O. Box 8299, Philadelphia, PA 19101), or other methods of birth control. The age of first sexual intercourse is a risk factor. Young, sexually experienced teenagers are three times more likely to have had PID.[21] The reason for this risk factor is not understood, but may be related to the fact that the younger patient is more likely to have multiple sexual partners, exposure to a larger pool of STDs, and larger areas of exposed endocervical epithelium.[22,23] Thus, it is extremely important to obtain an accurate and detailed sexual history from the patient, ideally when the patient is seen at the initial visit. If the patient states that she is not sexually active at the present time, the physician has an excellent opportunity to begin the educational process on transmission of STDs and the consequences of infection.

Patients older than 25 years who acquire a *C. trachomatis* or *N. gonorrhoeae* infection that leads to PID have a greater chance of resulting infertility than patients younger than 25 years.[24] However, the younger patient is more likely to experience repeated infection and episodes of PID. The patient who develops PID is at risk for tubal damage, depending upon the severity or degree of inflammation she experiences. Women with mild disease are unlikely to develop tubal damage, whereas women with severe disease are five times more likely to develop significant tubal damage.[25]

Once the patient has been given the facts concerning the transmission and consequences of STDs, the discussion should turn to contraceptive methods. Contraception should also be discussed with the patient, especially if sexual intercourse is occurring or is about to commence. Most individuals will have some knowledge of OCPs and perhaps Norplant. However, they will not be knowledgeable about the relationship of hormonal contraception and the acquisition of STDs. A look at the available data indicates that the use of oral contraceptives is associated with increased detection of cervical infection by *C. trachomatis*. However, oral contraceptives appear to be associated with a decreased risk of PID.[26–29] Although these findings are difficult to reconcile, they may be attributable to behavioral characteristics among individuals who use OCPs or to mild, undetected forms of PID among OCP users. There is also evidence that estrogen facilitates the adherence of *C. trachomatis* to endometrial cells. However, when estrogen is combined with high doses of progesterone, adherence is not favored.[30] Animal studies have not helped to clarify this issue. Oral contraceptives fed to guinea pigs seem to encourage both lower and upper genital tract infection by *C. trachomatis*.[31–34] In one study, the use of high-dose estrogen-containing pills appeared to be associated with a higher incidence of PID.[35]

Barrier contraception, including condoms and spermicides, offers enhanced protection against tubal infertility secondary to infection. The use of condoms alone provides only marginal protection. However, combining spermicides and condoms offers a two-fold increase in protection against tubal infertility.[35] This point should be emphasized to young women who are engaging in sexual intercourse but do not have reliable partners or whose partners have multiple partners.

Another risk factor, one which is poorly understood, is the relationship between cigarette smoking and the risk of tubal infertility. This relationship appears to be statistically stronger in nulliparous women and dependent upon the intensity of smoking. It does not appear to be related to behavioral patterns.[36–38] One hypothesis as to the mechanism by which smoking may be associated with tubal infertility is that smoking may interfere with the host immune response, thereby allowing the infecting organisms to gain entrance to the fallopian tube.[39] The relationship between exogenous, behavioral, and endogenous factors and the risk of tubal infertility is still unclear and remains to be unraveled.

**TABLE 1. Microbial flora of the healthy vagina**

| |
|---|
| Lactobacillus |
| Streptococcus |
| Staphylococcus |
| Corynebacterium |
| Diphtheroides |
| Enterococcus |
| Escherichia |
| Proteus |
| Klebsiella |
| Enterobacter |
| Morganella |
| Peptostreptococcus |
| Bacteroides |
| Fusobacterium |
| Veillonella |
| Eubacterium |

### MICROBIOLOGY AND INFERTILITY

The preceding discussion has alluded to the more common STDs and their role in infertility. The role of abnormal bacteriology of the lower genital tract has also been mentioned. The normal or healthy vaginal microflora is dominated by *Lactobacillus*, but numerous other bacteria make up the normal flora (Table 1). These bacteria exist in a dynamic relationship that is commensal, synergistic, and antagonistic. The bacteria also exist in a dynamic equilibrium with their environment, a balance that is constantly challenged by external and internal factors.

The relationship of these bacteria is dependent upon dominance which, in turn, is determined by bacterial metabolic products as well as environmental factors. In the healthy state, commensal bacteria, lactobacilli, corynebacteria, and diphtheroids are present in large numbers, with lactobacilli being present in concentrations of $>10^5$ cfu/cc of vaginal fluid. When the environmental conditions of the vagina become altered, the characteristics of the microbial flora are altered to favor the pathogenic organisms. This can happen when the pH is altered by environmental factors or by the introduction of exogenous pathogens. The most common organisms, e.g., *N. gonorrhoeae* and *C. trachomatis*, are introduced through sexual intercourse. However, other conditions not directly related to sexual activity may also have a bearing, for example, BV in which *T. vaginalis* and other organisms make up the microflora.

Bacteria such as *G. vaginalis* occupy a perplexing position in vaginitis. There is little doubt that this organism can play a pathogenic role under the appropriate set of circumstances. While there is considerable discussion over its role in vaginitis, there are no data to suggest that it plays a significant role in PID or infertility. Bacteria such as *Mycoplasma* and *Ureaplasma* occupy a significant controversial role in infertility.

Although the role of *Mycoplasma* and *Ureaplasma* as causative agents in infertility has not been established, they have been implicated. These bacteria have been associated with a variety of obstetrical and gynecologic infections, such as postpartum endometritis, postpartum bacteremia, post-cesarean abdominal wound infection, and early pregnancy loss.[40–44] Mycoplasmal organisms are consistently found in 20% to 40% of the healthy vaginal microbial flora, while colonization of the lower genital tract is directly related to the age of first coitus and the number of sexual partners.[45]

Infertility attributed to *Mycoplasma* is a subject that has waxed and waned over the years. In a study published in 1979, Gnarpe and Friberg divided 55 couples into four groups.[46] Group A consisted of 36 couples with unexplained infertility who had *Mycoplasma* colonization rates of 25% and *Ureaplasma* colonization rates of 92%. Group B contained 19 couples in which the females all had antisperm antibodies, 15% had *Mycoplasma*, and all had *Ureaplasma*. Group C was made up of 40 pregnant patients, of whom 7.5% had *Mycoplasma* and 22.5% had *Ureaplasma*. Group D consisted of 23 men with pregnant wives, none of whom had *Mycoplasma* but 26% of whom had *Ureaplasma*. This study suggested that *Mycoplasma* may play a role in infertility. Graber and colleagues studied 25 women with unexplained infertility and found *Ureaplasma* in 80% of these women compared to a 36% colonization rate in 25 fertile controls ($p < 0.001$).[47] Although this study did show a statistical correlation between *Mycoplasma* and infertility, a direct cause-and-effect relationship was not demonstrated. DeLouvois et al, in 1974, studied 120 infertile couples and 92 pregnant patients and found no statistical difference with regard to colonization rates of *Mycoplasma* or *Ureaplasma*.[45]

These studies encourage investigators to consider that, if *Mycoplasma* is involved in infertility, then treatment directed specifically against these bacteria should result in improved fertility rates. Friberg and Gnarpe sampled couples for the presence of *Mycoplasma*.[48] If the patients were positive, they were not treated for three months. If they did not achieve pregnancy within this time, both were treated with doxycycline, 100 mg twice daily for 7 to 16 days. They found a 25% pregnancy rate in 16 women having antisperm antibodies and a 29% pregnancy rate in 38 women not having antisperm antibodies. Although this study suggested that there may be a relationship between colonization and infertility and that treatment may be of benefit, there was no control group. Stray-Pedersen et al, in 1978, studied three groups of women.[49] Group A was made up of 46 women with a history of habitual abortion, 13 of whom were positive for *Ureaplasma*. All were treated with doxycycline and 13 who had positive cultures became pregnant and 10 delivered term infants. Group B consisted of 18 women with unexplained infertility, 50% of whom were colonized with *Ureaplasma*. Group C contained 45 fertile women, of whom only 7% were found to be colonized. In 1983, Toth et al reported on 161 infertile couples in whom only the males were found to be colonized with *Ureaplasma*.[50] They were treated with doxycycline, 100 mg twice daily for 28 days. Follow-up cultures showed that the organism had been eradicated in 129 patients. Sixty percent of the couples who were culture-negative achieved pregnancy, whereas only 5% of the couples in whom the male remained positive achieved pregnancy.

Several investigators have been unable to confirm the relationship of treating *Mycoplasma* and *Ureaplasma* colonization and achieving fertility. In 1975, Harrison et al. studied 88 couples and divided them into three groups.[51] Group A contained 30 couples with colonization rates of 23% for *Mycoplasma* and 70% for *Ureaplasma*. Group B contained 28 couples, of whom 14% were positive for

*Mycoplasma* and 64% for *Ureaplasma*. Group C consisted of 30 couples, of whom 26% were found to have *Mycoplasma* and 57% had *Ureaplasma*. Patients in group A were treated with doxycycline for 28 days and 16% achieved pregnancy. Individuals assigned to group B received a placebo, and 14% became pregnant. Group C received no treatment, neither doxycycline nor placebo, and 16% achieved pregnancy. Idriss et al, in 1978, studied 200 infertile couples and found that 42% of the treatment group became pregnant, whereas 37% of the nontreatment group became pregnant.[52]

Thus, the data regarding *Mycoplasma* and *Ureaplasma* are at best confusing. The studies that have been conducted lack a precise scientific approach. Perhaps studies should be carried out using quantitative bacteriology, serology, and DNA probes of the tissue in question. A patient with unexplained infertility is particularly perplexing because an explanation cannot be offered to her, creating a deeper sense of frustration in the patient. However, the development of new technology in the area of infectious disease is providing a better understanding of the subtleties of subclinical infection, which should permit a more scientific approach to studying the pathophysiology of tubal disease secondary to infection. This knowledge, in turn, will allow the clinician to prescribe a directed plan of management.

The most important aspect of preventing infertility or tubal damage secondary to infection is not the design of more potent treatment agents, but the prevention and recognition of the earliest signs of infection. Prevention of tubal damage and infertility does not begin with the treatment of salpingitis. Rather, the physician must begin the educational process on the patient's first visit. Dialogue should begin at this point and continue with each visit to reinforce what has been presented at earlier visits and keep the patient current on the latest information regarding transmission and protective measures. The second opportunity to prevent tubal damage and infertility is the recognition of the early signs of infection in the asymptomatic patient. These signs should be used to develop a significant history and to determine at what degree of risk the particular patient has placed herself.

### REFERENCES

1. Eschenback DA, Davick PR, Williams BL, et al: Prevalence of hydrogen peroxide-producing *Lactobacillus* species in normal women and women with bacterial vaginosis. J Clin Microbiol 27:251–256, 1989.
2. Berg AO, Heidrich FE, Fihn SN, et al: Establishing the cause of genitourinary symptoms in women in a family practice: Comparison of clinical examination and comprehensive microbiology. JAMA 251:620–625, 1984.
3. Fleury FJ: Adult vaginitis. Clin Obstet Gynecol 24:407–438, 1981.
4. Karnaky KJ: Diagnosis and treatment of vaginitis (letter). Am J Obstet Gynecol 129:929–930, 1977.
5. Fouts AC, Kraus SJ: *Trichomonas vaginalis:* Reevaluation of its clinical presentation and laboratory diagnosis. J Infect Dis 14:137–143, 1980.
6. Judson FN: The importance of coexisting syphilitic, chlamydial, mycoplasmal and trichomonal infection in the treatment of gonorrhea. Sex Transm Dis 6:112–119, 1979.
7. Wright RA, Judson FN: Relative and seasonal incidences of the sexually transmitted diseases. A two-year statistical review. Br J Vener Dis 54:433–440, 1978.
8. Lossick JG: The descriptive epidemiology of vaginal trichomoniasis and bacterial vaginosis. In Horowitz BJ, Mardh P-A (eds): Vaginitis and Vaginosis. New York, Wiley-Liss, p775, 1991.
9. Gardner HL, Dukes CD: *Haemophilus vaginalis* vaginitis. Am J Obstet Gynecol 69, 1955.
10. Faro S, Phillips LE: Non-specific vaginitis or vaginitis of undetermined aetiology. Int J Tissue React 9:173–177, 1987.
11. Faro S: Persistent vaginitis and vaginosis. In Horowitz BJ, Mardh P-A (eds): Vaginitis and Vaginosis. New York, Wiley-Liss, p 237, 1991.
12. Centers for Disease Control: Ectopic pregnancy—United States, 1988-1989. MMWR 41:591–594, 1992.
13. Cates W Jr, Rolfs RT Jr, Aral SO: Sexually transmitted diseases, pelvic inflammatory disease and infertility: An epidemiologic update. Epidemiol Rev 12:199–220, 1990.
14. Centers for Disease Control: Sexually transmitted disease surveillance. MMWR 1991.
15. Brunham RC, Paavonen J, Stevens CE, et al: Mucopurulent cervicitis: The ignored counterpart in women of urethritis in men. N Engl J Med 311:1–6, 1984.
16. Harrison HR, Costin M, Meder JB, et al: Cervical *Chlamydia trachomatis* infection in university women: Relationship to history, contraception, ectopy and cervicitis. Am J Obstet Gynecol 153:244–251, 1985.
17. Wasserheit JN, Bell TA, Kiviat NB, et al: Microbial causes of proven pelvic inflammatory disease and efficacy of clindamycin and tobramycin. Ann Intern Med 104: 187–193, 1986.
18. Paavonen J, Kiviat NB, Brunham RC, et al: Prevalence and manifestations of endometritis among women with cervicitis. Am J Obstet Gynecol 152:280–286, 1985.
19. Kiviat NB, Wolner-Hanssen P, Eschenbach DA, et al: Endometrial histopathology in patients with culture-proven upper genital tract infection and laparoscopically acute salpingitis. Am J Surg Pathol 14:167–175, 1990.
20. Paavonen J, Aine R, Teisala K, Heinonen PK, Punnonen R: Comparison of endometrial biopsy and peritoneal fluid cytologic testing with laparoscopy in the diagnosis of acute pelvic inflammatory disease. Am J Obstet Gynecol 151: 645–650, 1985.

21. Bell TA, Holmes KK: Age specific risks of syphilis, gonorrhea and hsopitalized pelvic inflammatory disease in sexually experienced U.S. women. Sex Transm Dis 11: 291–295, 1984.
22. Wasserheit JN: Pelvic inflammatory disease and infertility. Md Med J 36:58–63, 1987.
23. O'Reilly KR, Aral SO: Adolescents and sexual behavior: Trends and implications for STD. J Adolesc Health Care 6:262–270, 1985.
24. Westrom K, Mardh P-A: Acute pelvic inflammatory disease (PID). In Holmes KK, Mardh P-A, Sparling PF, et al. (eds): Sexually transmitted diseases. 2nd ed. New York: McGraw-Hill, p 593, 1990.
25. Westrom L: Effect of acute pelvic inflammatory disease on fertility. Am J Obstet Gynecol 121:707–713, 1975.
26. Washington AE, Gove S, Schachter J, et al: Oral contraceptives, *Chlamydia trachomatis* infection, and pelvic inflammatory disease. JAMA 253:2246–2250, 1985.
27. Senanayake P, Kramer DG: Contraception and the etiology of pelvic inflammatory disease: New perspectives. Am J Obstet Gynecol 138:852–860, 1980.
28. Svensson L, Mardh P-A, Westrom L: Infertility after acute salpingitis with special reference to *Chlamydia trachomatis*. Fertil Steril 40:322–329, 1983.
29. Wolner-Hanssen P, Eschenbach DA, Paavonen J, et al: Decreased risk of symptomatic chlamydial pelvic inflammatory disease associated with oral contraceptive use. JAMA 251:2553, 1984.
30. Maslow AS, Davis CH, Choong J, Wyrick PB: Estrogen enhances attachment of *Chlamydia trachomatis* to human endometrial epithelial cells in vitro. Am J Obstet Gynecol 159:1006–1014, 1988.
31. Barron AL, Pasley JN, Rank RG, White HJ, Mrak RE: Chlamydia salpingitis in female guinea pigs receiving oral contraceptives. Sex Transm Dis 15:169–173, 1988.
32. Rank RG, White HJ, Hough AJ, Pasley JN, Barron AL: Effect of estradiol on Chlamydial infection in female guinea pigs. Infect Immun 38:699–705, 1982.
33. Rank RG, Barron AL: Specific effects of estradiol on genital mucosal antibody response in chlamydial ocular and genital infections. Infect Immun 55:2317, 1987.
34. Pasley JN, Rank RG, Hough AJ, et al: Absence of progesterone effects on chlamydial genital infection in female guinea pigs. Sex Transm Dis 12:155–158, 1985.
35. Cramer DW, Goldman MB, Schiff I, et al: The relationship of tubal infertility to barrier method and oral contraceptive use. JAMA 257:2446–2450, 1987.
36. Daling JR, Weiss NS, Spadoni LR, et al: Cigarette smoking and primary tubal infertility. In Rosenbert MJ (ed): Smoking and reproductive health. Littleton MA: PSG Publishing, p 40, 1987.
37. Phipps WR, Cramer DW, Schiff I, et al: The association between smoking and female infertility as influenced by cause of the infertility. Fertil Steril 48:377–382, 1987.
38. Marchbanks PA, Lee NC, Peterson HB: Cigarette smoking as a risk factor for pelvic inflammatory disease. Am J Obstet Gynecol 162:639–644, 1990.
39. Hershey P, Pendergast D, Edwards A: Effects of cigarette smoking on the immune system: Follow-up studies in normal subjects after cessation of smoking. Med J Aust 2:425, 1983.
40. Phillips LE, Faro S, Pokorny SF, et al: Postcesarean wound infection by *Mycoplasma hominis* in a patient with persistent postpartum fever. Diag Microbiol Infect Dis 7:193–197, 1987.
41. Faro S, Martens M, Hammill H, Phillips LE, Smith D, Riddle G: Ticarcillin/clavulanic acid versus clindamycin and gentamicin in the treatment of post-cesarean endometritis following antibiotic prophylaxis. Obstet Gynecol 73:808–812, 1989.
42. Maccato M, Faro S, Summers KL: Wound infections after cesarean section with *Mycoplasma hominis* and *Ureaplasma urealyticum:* A report of three cases. Diagn Microbiol Infect Dis 13:363–365, 1990.
43. Roberts S, Maccato M, Faro S, Pinell P: The microbiology of post-cesarean wound morbidity. Obstet Gynecol 81:383–386, 1993.
44. Kundsin RB, Driscoll SG, Ming PL: Strain of *Mycoplasma* associated with human reproductive failure. Science 157:1573–1574, 1967.
45. Louvois J de, Blades M, Harrison RF, Hurley R, Stanley VC: Frequency of *Mycoplasma* in infertile and fertile couples. Lancet 1:1073–1075, 1974.
46. Gnarpe H, Friberg J: *Mycoplasma* and human reproductive failure. I. The occurrence of different mycoplasmas in couples with reproductive failure. Am J Obstet Gynecol 114:727–731, 1972.
47. Graber CD, Creticos P, Valicdenti J, Williamson HO: *T-mycoplasma* in human reproductive failure. Obstet Gynecol 54:558, 1979.
48. Friberg J, Gnarpe H: *Mycoplasma* and human reproduction. III. Pregnancy in infertile couples treated with doxycycline for *T-mycoplasma*. Am J Obstet Gynecol 116:23, 1973.
49. Stray-Pedersen B, Eng J, Reikvam TM: Uterine *T-mycoplasma* colonization in reproductive failure. Am J Obstet Gynecol 130:307–311, 1978.
50. Toth A, Lesser WL, Brooks C, Labriola D: Subsequent pregnancy in 161 couples treated for *T-mycoplasma* genital tract infection. N Engl J Med 308:505–507, 1983.
51. Harrison RF, Blades M, DeLouvois J, Hurley R: Doxycycline treatment and human fertility. Lancet 1:605, 1975.
52. Idriss WM, Patton WC, Taymor MC: On the etiologic role of *Ureaplasma* urealyticum (*T-mycoplasma*) infection in infertility. Fertil Steril 30:293–296, 1978.
